# The Association Between Diabetic Retinopathy and the Prevalence of Age-Related Macular Degeneration—The Kailuan Eye Study

**DOI:** 10.3389/fpubh.2022.922289

**Published:** 2022-07-18

**Authors:** Zhang Yongpeng, Wang Yaxing, Zhou Jinqiong, Wang Qian, Yan Yanni, Yang Xuan, Yang Jingyan, Zhou Wenjia, Wang Ping, Shen Chang, Yang Ming, Luan Yanan, Wang Jinyuan, Wu Shouling, Chen Shuohua, Wang Haiwei, Fang Lijian, Wan Qianqian, Zhu Jingyuan, Nie Zihan, Chen Yuning, Xie Ying, Jost B. Jonas, Wei Wenbin

**Affiliations:** ^1^Beijing Key Laboratory of Intraocular Tumor Diagnosis and Treatment, Beijing Ophthalmology and Visual Sciences Key Lab, Medical Artificial Intelligence Research and Verification Key Laboratory of the Ministry of Industry and Information Technology, Beijing Tongren Eye Center, Beijing Tongren Hospital, Capital Medical University, Beijing, China; ^2^Beijing Key Laboratory of Ophthalmology and Visual Sciences, Beijing Tongren Eye Center, Beijing Institute of Ophthalmology, Beijing Tongren Hospital, Capital Medical University, Beijing, China; ^3^Cardiology Department, Kailuan General Hospital, Tangshan, China; ^4^Health Care Center, Kailuan Group, Tangshan, China; ^5^Department of Ophthalmology, Fuxing Hospital, Capital Medical University, Beijing, China; ^6^Department of Ophthalmology, Beijing Liangxiang Hospital, Capital Medical University, Beijing, China; ^7^Department of Ophthalmology, The Second Hospital of Anhui Medical University, Hefei, China; ^8^Department of Ophthalmology, Shanxi Provincial People's Hospital, Taiyuan, China; ^9^Department of Ophthalmology, Medical Faculty Mannheim of the Ruprecht-Karls-University of Heidelberg, Heidelberg, Germany

**Keywords:** diabetes mellitus, diabetic retinopathy, age-related macular degeneration, prevalence, epidemiology

## Abstract

This study aimed to investigate the prevalence of age-related macular degeneration (AMD) in patients with diabetes mellitus (DM) and diabetic retinopathy (DR) and analyze whether DR is a risk factor for AMD. This population-based epidemiological study included 14,440 people from the Kailuan Eye Study in 2016, of whom 1,618 were patients with type 2 DM aged over 50 years, and 409 had DM with DR. We analyzed whether there were differences in the prevalence of AMD between DM with DR and DM without DR, and conducted a hierarchical statistical analysis according to different stages of DR. Using variable regression analysis, we explored whether DR constituted a risk factor for AMD. In the DM population, the prevalence of wet AMD in patients with DM with and without DR was 0. 3 and 0.2%, respectively, with no significant difference (*P* = 0.607). Meanwhile, the prevalence of dry AMD in patients with DM with and without DR was 20.8 and 16.0%, respectively, with a significant difference. In the subgroup analysis of dry AMD, the prevalence of early, middle, and late dry AMD in DM with DR was 14.4, 5.9, and 0.5%, respectively. In DM without DR, the prevalence of early, middle, and late dry AMD was 10.5, 4.8, and 0.7%, respectively (*P* = 0.031). In the subgroup analysis of DR staging, statistical analysis could not be performed because of the limited number of patients with PDR. In the variable regression analysis of risk factors for dry AMD, after adjusting for age, sex, body mass index, hypertension, and dyslipidemia, DR constituted the risk factor for dry AMD. In conclusion, DM did not constitute a risk factor for AMD, and the prevalence of wet AMD and dry AMD in patients with DM and DR was higher than that in patients with DM without DR (among which dry AMD was statistically significant). Multivariate regression analysis confirmed that DR is an independent risk factor for dry AMD. Reasonable control of DM and slowing down the occurrence and development of DR may effectively reduce the prevalence of AMD in patients with DM.

## Introduction

Diabetes mellitus (DM), a metabolic disorder, can affect multiple systems of the body. The eye is an important organ that can be affected by DM, and almost every ocular structure may be involved. Diabetic retinopathy (DR), which seriously affects visual function, is associated with the progression of DR and DM. The prevalence of DR increases with the duration of DM. After 10–15 years from the diagnosis of DM, patients develop DR (1). The prevalence of DM has increased in both developing and developed countries (1).

In China, the prevalence of DM increased from <1% in 1980 to 11.6% in 2013, with 114 million people affected (2). As a major complication of DM, DR is the leading cause of blindness in the working-age population. The duration of DM and glycemic control levels have a major effect on the development of DM complications. The prevalence of DR increases with the aging of the Chinese population. In the adult DM population in northern China, the prevalence of DR is as high as 37.1–43.1%, that is, more than one-third of patients with DM have DR, of which the prevalence of DR threatening vision is 5–6.3%. As an irreversible blinding eye disease, DR is a heavy burden on society and families (3–6).

However, more attention should be paid to age-related macular degeneration (AMD). AMD can be dry (non-exudative) or wet (exudative). The late stages of AMD include geographic atrophy (GA) and wet AMD. Globally, AMD is the leading cause of blindness in people over 50 years of age. Moreover, 8.7% of the worldwide population has AMD, and the projected number of people with the disease is approximately 196 million in 2020 and 288 million in 2040 (7, 8). Epidemiological studies on the prevalence of AMD in northern China show that the prevalence of early AMD is 1.4%-3.0%, and that of late AMD is 0.1–0.2% (9–11). The treatment of advanced AMD is difficult and expensive, placing a heavy economic burden on families and society.

As the country with the largest population in the world, China has begun to enter an aging society. Visual quality is an important aspect of improving the sense of acquisition of quality of life in the elderly. DR and AMD, two important blinding eye diseases, seriously affect the quality of life of patients, and their prevention and treatment are urgently needed. Patients with DM will eventually reach the age group of more than 50 years. What are the results when the two diseases are superimposed? The conclusions drawn from different studies were controversial. Some studies have shown that DM and DR can increase the prevalence of AMD (12–21), whereas some have shown that DM and DR are not related to the onset of AMD (22–27) and that DM or DR might be protective factors against AMD (28–32). The research methods adopted, such as epidemiological research, medical insurance data, case-control, sampling population, age setting, disease classification, and staging standards, the number of cases, and race, may have affected the differences in research conclusions.

This population-based epidemiological study in northern China aimed to explore the relationship between the prevalence of AMD in DR and non-DR populations and provide a scientific basis for the comprehensive prevention and control of the two ocular diseases with a high blindness rate.

## Materials and Methods

The Kailuan Eye Study was introduced previously (33–36). This retrospective cross-sectional study included 14,440 people who had undergone ophthalmologic and general examinations from the longitudinal Kailuan Study in 2016.

The body mass index (BMI) was calculated for all study participants. BP was assessed with the participants sitting for at least 5 min. Blood samples were collected under fasting conditions to determine the blood glucose, high-density lipoprotein cholesterol (HDL-C), low-density lipoprotein cholesterol (LDL-C), triglyceride (TC), and total cholesterol (TG) concentrations.

Ophthalmological examinations included measurement of visual acuity, intraocular pressure, and slit-lamp-assisted biomicroscopy of the eye. Using a non-mydriatic fundus camera (CR6-45 NM; Canon, Inc, Osta, Tokyo, Japan), we obtained two 45° fundus photographs centered on the optic nerve head and macula. If the pupil diameter did not allow fundus photography with sufficient photographic quality, the pupil was dilated medically by applying eye drops containing 0.5% tropicamide and 0.5% phenylephrine hydrochloride. Using fundus photographs, DR was assessed in a masked manner without knowledge of other ocular or systemic parameters of the study participants. Both eyes were evaluated.

The diagnosis of DM was based on any of the following three criteria: measurement of the fasting blood glucose concentration of 7.0 mM, a self-reported history of DM, or a history of medication with hypoglycemic agents.

The diagnostic criteria for hypertension were blood pressure of ≥140/90 mmHg, positive history of hypertension, or the use of antihypertensive drugs.

The clinical classification of hyperlipidemia included hypercholesterolemia (TC≥6.2 mmol/L), hyperglycemia (TG≥2.3 mmol/L), mixed hyperlipidemia (TC ≥6.2 mmol /L and TG≥2.3 mmol/L), and low HDL-C levels (<1.0 mmol/L).

The diagnostic criteria for BMI were based on the guidelines for prevention and control of overweight and obesity in Chinese adults as follows: underweight (BMI, <18.5 kg/m^2^), normal (BMI, 18.5–23.9 kg/m^2^), overweight (BMI, 24.0–27.9 kg/m^2^), obese (BMI, ≥28 kg/m^2^).

The diagnostic criteria for AMD were based on the Beckman Macular Research Classification System. The lesions were assessed within two optic disc diameters of the fovea of either eye. AMD was classified as follows: no apparent aging changes (no drusen and no AMD pigmentary abnormalities), normal aging changes (small drusen of ≤ 63γm and no AMD pigmentary abnormalities), early AMD (medium drusen of > 63 γm, ≤ 125 γm, and no AMD pigmentary abnormalities), intermediate AMD (large drusen of >125 γm or any AMD pigmentary abnormalities), and late AMD (neovascular AMD or any geographic atrophy).

DR grading was performed according to the Early Treatment of Diabetic Retinopathy Study (ETDRS) criteria. DR severity was graded as mild non-proliferative DR (20 ETDRS levels of <43 with at least one microaneurysm), moderate non-proliferative DR (43 ETDRS levels of <53), severe non-proliferative DR (53 ETDRS levels of <61), and proliferative DR (ETDRS level of 61). An experienced and trained ophthalmologist assessed the photographs. In cases of doubt, photographs were reassessed by a panel that included several ophthalmologists.

Statistical analysis was performed using the R software. The results were expressed as mean and standard deviation or as mean and 95% CI. Logistic regression models were used to estimate the odds ratios (ORs) and their 95% CIs for each risk factor for DR. Statistical significance was set at *P* < 0.05.

### Ethics Statement

This study adhered to the tenets of the Declaration of Helsinki. The Medical Ethics Committee of Beijing Tongren Hospital approved the study protocol, and informed consent was obtained from all patients after an explanation of the nature and possible consequences of the study.

## Results

**Figure 1:**
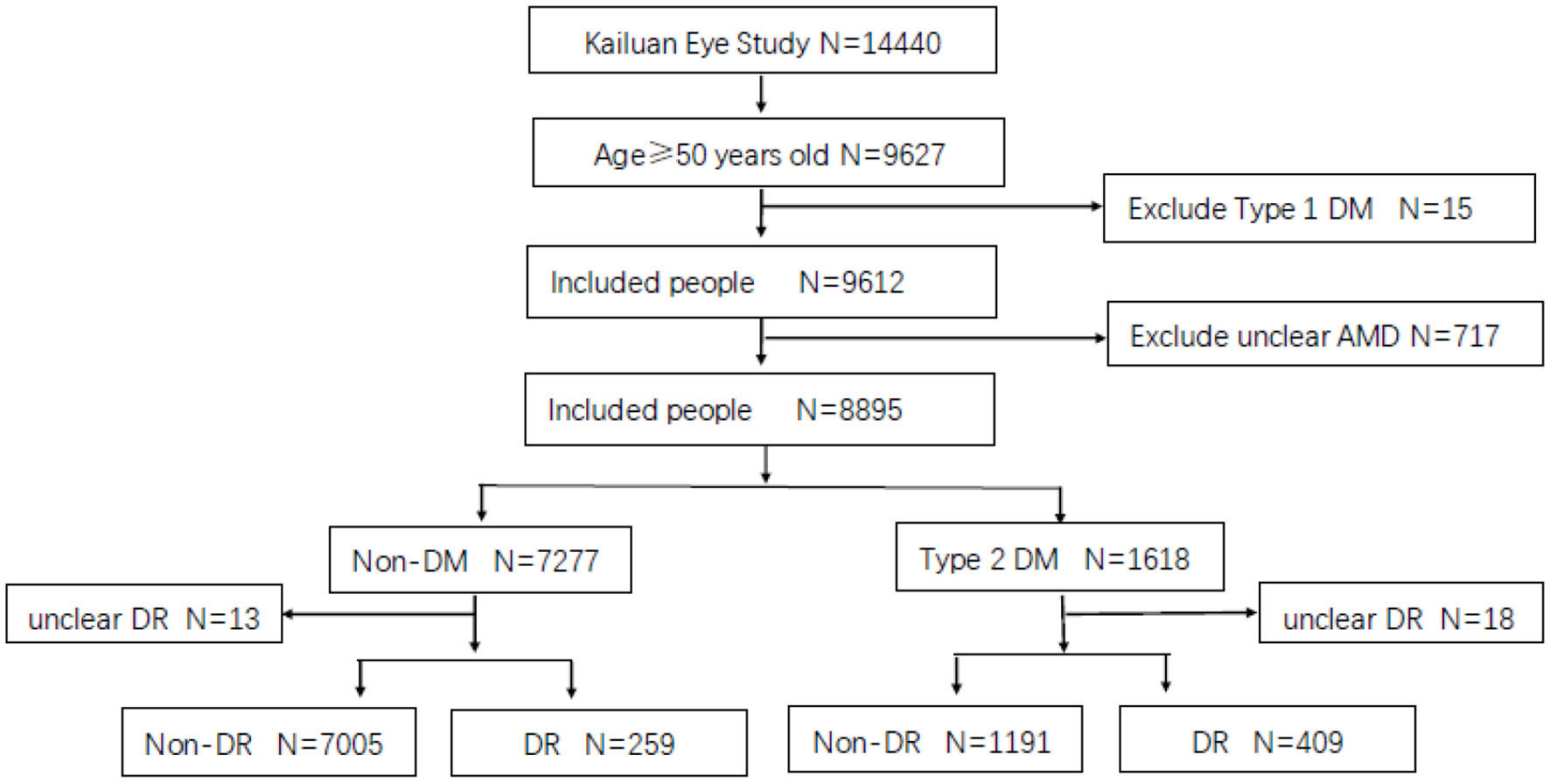
Flow chart of included and excluded population.

A total of 14,440 participants were enrolled in the Kailuan Eye Study, of whom 9,627 were older than 50 years old. Fifteen were excluded for having type 1 DM and 717 for an unclear diagnosis of AMD. Finally, 8,895 were included in the analysis group. Among them, the number of patients with type 2 DM was 1,618, 409 of whom had DM combined with DR, and 1,191 had DM without DR, excluding 18 people diagnosed with unclear DR. The prevalence of DM aged 50 years was 16.83%. The prevalence of DR was 25.28% in the population with DM aged > 50 years (Figure 1).

Notably, among the 7,277 non-DM participants, 13 were excluded from image unrecognition, and 259 people were found to have DR image characteristics, including microaneurysm, retinal hemorrhage, hard exudation, cotton–wool, and retinal neovascularization, in the non-DM group, with a prevalence of 3.56%. There are two possibilities; one is that in epidemiological field screening, although the random blood glucose level is normal, there may be abnormal glucose tolerance or low insulin function; and another reason is that the current diagnostic criteria for DM are not suitable for this group of people; that is, the blood glucose level for the normal population is already high.

### The Prevalence of AMD in Diabetic and Non-DM Participants

The prevalence of wet and dry AMD was 0.5% and 16.4%, respectively, in the non-DM population (7,277 people). In the patients with type 2 DM (1,618 people), the prevalence of wet AMD and dry AMD was 0.3 and 17%, respectively. There was no significant difference in the prevalence of wet and dry AMD between the diabetic and non-diabetic participants.

In a staging study of dry AMD, the prevalence of dry AMD in the early, middle, and late stages without DM was 11.7, 4.4, and 0.3%, respectively. The prevalence of dry AMD in the early, moderate, and late stages of DM was 11.4, 5, and 0.6%, respectively, with no significant differences (Table 1).

**Table 1:** The prevalence of AMD in diabetic and non-diabetic people.

	**Wet AMD** (%)^*^	**Dry AMD** (%)^**^	**Dry AMD (*****n*** **=** **1,469)**		
			**Early dry AMD (%)**	**Middle dry AMD (%)**	**Late dry AMD (%)**
Non-DM *n* = 7,277	33 (0.5)	1,194 (16.4)	850 (11.7)	321 (4.4)	23 (0.3)
DM *n* = 1,618	5 (0.3)	275 (17.0)	184 (11.4)	81 (5.0)	10 (0.6)
Total *n* = 8,895	38	1,469	1,034	402	33

* *χ^2^ = 0.354, P = 0.551. When test level α = 0.05, there is no difference of the prevalence of wet AMD between diabetic and non-diabetic people*.

* **χ^2^ = 0.291, P = 0.590. When test level α = 0.05, there is no difference of the prevalence of dry AMD between diabetic and non-diabetic people.

*AMD, age-related macular degeneration*.

### The Prevalence of AMD in DM With DR and DM Without DR

In the population with DM without DR (1191 people), the prevalence of wet AMD and dry AMD was 0.3 and 16.0%, respectively. In the population of patients with DM and DR (409 people), the prevalence of wet and dry AMD was 0.5 and 20.8%, respectively. The Fisher's exact probability method showed that there was no significant difference in the prevalence of wet AMD between the DR and non-DR groups (*P* = 0.607 and α = 0.05), whereas there was a significant difference in the prevalence of dry AMD between the DR and non-DR groups when α = 0.05 (χ^2^ = 4.655 and *P* = 0.031). The prevalence of dry AMD was higher in the DR group than in the non-DR group.

In a staging study of dry AMD in the non-DM population, the prevalence of early, middle, and late dry AMD was 10.5, 4.8, and 0.7%, respectively, in the DM population, the prevalence of early, middle, and late dry AMD was 14.4, 5.9, and 0.5%, respectively, with significant differences (Table 2).

**Table 2:** The prevalence of AMD in DM with DR and DM without DR.

	**Wet AMD** (%)^*^	**Dry AMD** (%)^**^	**Dry AMD (*****n*** **=** **275)**		
			**Early dry AMD (%)**	**Middle dry AMD (%)**	**Late dry AMD (%)**
Non-DR *N* = 1,191	3 (0.3)	190 (16.0)	125 (10.5)	57 (4.8)	8 (0.7)
DR *n* = 409	2 (0.5)	85 (20.8)	59 (14.4)	24 (5.9)	2 (0.5)
Total *n* = 1,600	5	275	184	81	10

* *Fisher P = 0.607, when test level α = 0.05, the prevalence of wet AMD in DR and non-DR is not statistical significance*.

** *χ^2^=4.655, P=0.031, when test level α=0.05, the prevalence of dry AMD in DR is higher than that of non-DR with statistical significance*.

*AMD, age-related macular degeneration; DR, diabetic retinopathy; DM, diabetes mellitus*.

### The Prevalence of AMD in Different DR Stages of DM

In different stages of DM, because wet AMD did not combine with moderate NPDR (*n* = 66) and severe NPDR (*n* = 2), it was impossible to calculate the difference in the prevalence of dry and wet AMD (Table 3).

**Table 3:** The prevalence of AMD in different stages of DR with DM.

	**Wet AMD (%)**	**Dry AMD (%)**	**Dry AMD (n=85)**		
			**Early dry AMD (%)**	**Middle dry AMD (%)**	**Late dry AMD (%)**
Mild NPDR *n* = 341	2 (0.6)	77 (22.6)	54 (15.8)	22 (6.5)	1 (0.3)
Moderate NPDR *n* = 66	0 (0.0)	8 (12.1)	5 (7.6)	2 (3.0)	1 (1.5)
PDR *n* = 2	0 (0.0)	0 (0.0)	0 (0.0)	0 (0.0)	0 (0.0)
Total *n* = 409	2	85	59	24	2

*AMD, age-related macular degeneration; DR, diabetic retinopathy, DM, diabetes mellitus*.

### The Prevalence of AMD in Different “DR” Stages of Non-DM

In non-DM participants, some patients with “DR” were also staged according to DR. Because moderate NPDR (*n* = 44), severe NPDR (*n* = 1), and PDR (*n* = 1) were not combined with wet AMD, it is impossible to count the difference in the prevalence of dry and wet AMD in the different “DR” stages of non-DM (Table 4).

**Table 4:** The prevalence of AMD in different stages of “DR” with non-DM.

	**Wet AMD (%)**	**Dry AMD (%)**	**Dry AMD (*****n*** **=** **57)**		
			**Early dry AMD (%)**	**Middle dry AMD (%)**	**Late dry AMD (%)**
Mild NPDR *n* = 216	1 (0.5)	48 (22.2)	30 (13.9)	16 (7.4)	2 (0.9)
Moderate NPDR *n* = 41	0 (0.0)	9 (22.0)	5 (12.2)	4 (9.8)	0 (0.0)
Severe NPDR *n* = 1	0 (0.0)	0 (0.0)	0 (0.0)	0 (0.0)	0 (0.0)
PDR *n* = 1	0 (0.0)	0 (0.0)	0 (0.0)	0 (0.0)	0 (0.0)
Total *n* = 259	1	57	35	20	2

*AMD, age-related macular degeneration; DR, diabetic retinopathy; DM, diabetes mellitus*.

### Multivariate Analysis of Influencing Factors of Dry AMD

In the analysis of risk factors for dry AMD, the following three models were established: Model 1, corrected age; Model 2, corrected for age, sex, and BMI (< 8.5, 18.5–23.9, 24–27.9, ≥ 28); and Model 3, adjusted for age, sex, BMI, hypertension (yes or no), and dyslipidemia (yes or no).

The results showed that DR is a risk factor for dry AMD, and male sex and age were risk factors for dry AMD. Meanwhile, BMI, hypertension, and hyperlipidemia were not identified as risk factors for dry AMD (Table 5).

**Table 5:** The risk factor for dry AMD (*n* = 8,857).

	**Model 1 OR (95% CI)^**a**^**	**Model 2 OR (95% CI)^**b**^**	**Model 3 OR (95% CI)^**c**^**
Ref=non-DR			
DR	1.35 (1.05, 1.72)	1.38 (1.07, 1.78)	1.38 (1.07, 1.78)
Ref=female			
Male	1.33 (1.17, 1.52)	1.32 (1.13, 1.55)	1.33 (1.13, 1.56)
Ref=lower than 60 years old			
60–69 years old	1.62 (1.43, 1.84)	1.62 (1.40, 1.89)	1.62 (1.39, 1.89)
≥70 years	2.11 (1.79, 2.50)	2.36 (1.90, 2.92)	2.35 (1.89, 2.92)
Ref=normal weight (BMI: 18.5–23.9)			
Low weight (BMI: <18.5)	-	0.57 (0.26, 1.10)	0.57 (0.26, 1.10)
Overweight (BMI: 24.0–27.9)	-	0.95 (0.82, 1.11)	0.95 (0.81, 1.10)
Obesity (BMI:≥28.0)	-	1.12 (0.92, 1.37)	1.10 (0.90, 1.35)
Ref=non hypertension			
Hypertension	-	-	1.01 (0.88, 1.17)
Ref=non hyperlipidemia			
Hyperlipdemia	-	-	1.11 (0.95, 1.31)

a *Model 1: corrected age (50–59, 60–69, ≥70) and gender (male, female)*.

b *Model 2: corrected age, gender, and BMI (<18.5, 18.5–23.9, 24–27.9, ≥28)*.

c *Model 3: corrected age, gender, BMI, hypertension (yes or no), hyperlipidemia (yes or no)*.

*AMD, age-related macular degeneration; DR, diabetic retinopathy; DM, diabetes mellitus*.

## Discussion

The global crude prevalence of avoidable vision impairment and blindness in adults aged ≥ 50 years did not change between 2010 and 2019 (37). The leading global cause of blindness in those aged 50 years and older in 2020 was cataracts, followed by glaucoma, under-corrected refractive error, AMD, and DR (37). Although DR was the fifth leading cause of blindness in 2020, it was the only cause of blindness that showed a global increase in age-standardized prevalence between 1990 and 2020. Furthermore, more than 600 million people are projected to live with diabetes by 2040 (38). Because people with DM live increasingly longer with improvements in medical conditions, the number of people with DR and vision impairment is expected to increase rapidly, especially in China. DR management requires a disproportionate amount of social and economic resources. The prevalence of blindness due to AMD has declined by almost 30% from 1990 to 2020. This decrease is probably associated with the widespread clinical introduction of anti-VEGF therapy for wet AMD. However, most patients with AMD have untreatable dry AMD that can progress to GA (37). DR and AMD remain the most important ocular public health problems in China and worldwide.

International Diabetes Federation (IDF) estimated the global population with DM to be 463 million in 2019 and projected it to be 700 million by 2045 (2). As the most common and specific complication of DM, DR is one of the leading causes of preventable blindness in the adult working population (39). A declining trend in DR prevalence has been suggested, particularly in developed countries (40–42). This is the result of systemic control in patients with DM. It has been estimated that 51% of all the patients with blindness due to DR come from the Asia-Pacific region. The prevalence of DR among patients with DM ranges from 10% in India to 43% in Indonesia within the Asia-Pacific (43, 44). The highest number of people with DM is in China (116 million), which reflects the rapid economic growth and urbanization in China with significant lifestyle and dietary changes (39). In China, a large-scale population-based study conducted on 46,239 adults reported a 14-fold increase in DM prevalence over 30 years (0.67% in 1980 vs. 9.7% in 2008) (45).

Globally, AMD is the leading cause of blindness among people over 50 years old (7, 8). Epidemiological studies on the prevalence of AMD in northern China show that the prevalence of early AMD is 1.4–3.0%, and that of late AMD is 0.1–0.2% (9–11). The prevalence of early AMD in the Chinese sample was similar to that in white individuals in the Blue Mountains Eye Study (BMES) and other studies (10).

Today's working-age group will become tomorrow's elderly group. What is the relationship between DM, DR, and AMD in the Chinese population? Some research has been conducted on this topic, but the conclusion is contradictory.

The research methods on the relationship between DM or DR and AMD include longitudinal studies (prospective cohort study and retrospective cohort study), cross-sectional studies (cross-sectional population-based study and observational analysis of randomized clinical trial), case-control studies (retrospective descriptive observational case-control and case-control studies), and systematic reviews and meta-analyses. The study design that we used was cross-sectional population-based epidemiology. Every method has its advantages but also has limitations. In this study, AMD included early, middle, and late stages, and late-stage AMD included GA and wet AMD. DR includes NPDR and PDR, whereas NPDR includes mild, moderate, and severe NPDR. It is difficult to correlate each stage of DR to each stage and type of AMD individually, which requires epidemiological research with a large sample size and long-term longitudinal follow-up.

The relationship between DR and AMD remains inconsistent because of different research methods and sample sizes; that is, some studies have shown that DM and DR can increase the prevalence of AMD (12–21), some have shown that DM and DR are not related to AMD (22–27), and some have shown that DM or DR might protect against AMD (28–32).

Early studies did not find a relationship between DM and AMD (12–21). Later, the Beaver Dam Eye Study (BDES) (17) and BMES (15) found that DM and AMD were correlated, but their conclusions were contradictory. The BDES showed that DM was not associated with early AMD or GA. However, the prevalence of exudative AMD among people with DM was higher (9.4%) than that of those without DM (4.7%) who were older than 75 years. The BMES showed that DM was only associated with GA but not with wet AMD or early AMD. In 2013, Medicare data from 6,621 patients over the age of 69 years with newly diagnosed DM (1995–2005) in the United States showed that NPDR significantly increased the risk of dry AMD and wet AMD, whereas PDR can only increase the risk of wet AMD. The risk of dry AMD and wet AMD did not increase in patients with DM, but not without DR. There was no difference in the risk of wet AMD between PDR and NPDR (12). A retrospective study of the longitudinal health insurance database of Taiwan (1997–2012) showed that patients with DM had a 1.4-fold increase in dry and wet AMD compared to matched patients without DM, although the difference was not significant. In contrast, patients with DM and DR had a 4 and 3.9-fold increased incidence of dry and wet AMD, respectively. The HR for the development of dry AMD and wet AMD was 3.89 and 3.42 for patients with DM and DR and those without DR, respectively (*P* < 0.001) (14). This is consistent with the conclusion of our study that DM may not be a risk factor for increasing the prevalence of AMD, but DR does.

However, studies have shown that the incidence rate of AMD in the DM population is lower than that in the general population (28–31). Recent research has shown that the prevalence of neovascular AMD of the eyes in people with DR is low (0.04%). Moreover, the prevalence of choroidal neovascularization (CNV) in eyes with DR is low, with a lower prevalence of AMD. Diabetic choroidopathy plays a significant role in CNV formation in eyes with DR (32).

The findings of our study showed that DM had no effect on the prevalence of AMD, but DR could promote the development of dry AMD. The prevalence of wet AMD and dry AMD was 0.5 and 16.4%, respectively, in the non-DM population and 0.3 and 17%, respectively, in the DM population. There was no significant difference in the prevalence of wet and dry AMD between the diabetic and non-diabetic individuals.

The prevalence of wet AMD and dry AMD was 0.3% and 16.0%, respectively, in the population with DM without DR and 0.5 and 20.8%, respectively, in the population with DM and DR. There was no significant difference in the prevalence of wet AMD between the DR and non-DR groups. The prevalence of dry AMD was significantly higher in the DR group than in the non-DR group.

In the Kailuan Eye Study, we also found another finding of “DR” in the non-DM population. Among the 7,277 non-DM subjects, 259 people were found to have “DR” image characteristics, including microaneurysm, retinal hemorrhage, hard exudation, cotton-wool, and retinal neovascularization in no-DM, and the prevalence was 3.56%. There have been reports of retinopathy in persons without DM in the Handan Eye Study (46). The prevalence of retinopathy among participants without DM was 13.6%, and the age and sex-standardized prevalence of retinopathy in the Chinese adult population (aged 30 years) without DM was estimated to be 12.1%. Its association with fasting plasma glucose and BP suggests that early microvascular damage occurs at “high normal” levels of blood.

Why can DR accelerate the onset of AMD? DR may share common pathogenic pathways with AMD. The biological interplay between DR and AMD is complicated and not fully understood (13). First, DM may lead to the accumulation of highly stable advanced glycation end products (AGEs) in multiple tissues, including the retinal pigment epithelium (RPE) cell layers and photoreceptors, which are implicated in the pathogenesis of AMD (47–50). Second, hyperglycemia and dyslipidemia in patients with disturbed homeostasis of the retina by inducing inflammatory responses, which might play a role in AMD (51–53). Third, VEGF plays an important role in both DR and AMD, and both benefit from anti-VEGF treatment (13, 14). Fourth, swept-source optical coherence tomography demonstrated a significant reduction in central macular thickness in PDR compared to controls (54). Understanding diabetic choroidopathy may improve our knowledge of the mechanisms underlying DR and AMD. Finally, structural retinal changes occur with aging. Retinal thickness decreases with age, especially in the inner nuclear layer. The macular blood flow was reduced by an average of 20%. Microglia are less motile and respond more slowly to injury. RPE decreases melanin production and increases lipofuscin accumulation. Müller cells are more susceptible to oxidative stress (55). These anatomical changes may contribute to the pathogenesis of DR combined with AMD in the elderly population.

DR is a major complication of DM. Globally, DR is the leading cause of preventable blindness in adults aged 20–74 years. Because it is avoidable blindness, slowing down the occurrence of DR after the occurrence of DM should be the key point. In most patients, retinopathy develops 10–15 years after the diagnosis of DM (1). The most common modifiable risk factors for DR in the Asia–Pacific region are hyperglycemia, BP, dyslipidemia, and obesity (43). Data from the UK Prospective Diabetes Study (UPDS) and the Diabetes Control and Complications Trial (DCCT) showed that controlling blood glucose (glycated hemoglobin A1c, HbA1c <7%) and BP levels slowed the onset and progression of DR (56–59). In China, a 5-year community-based prospective study on patients with type 2 DM showed DR regression in 24% of patients, which occurred mostly in patients with lower glucose levels (60). In Hong Kong, the regression rate was 13.2% and was also associated with lower HbA1c levels and the absence of albuminuria (61). These data suggest that the onset and progression of DR may be slowed down by reasonably controlling DM to reduce the prevalence of AMD in patients with DM.

The limitation of this study is its cross-sectional design rather than a longitudinal study. Therefore, the prevalence can only be discussed, and its incidence cannot be analyzed. Although our study was a population-based epidemiological study, the sample size was limited. Some diseases require hierarchical research, and some studies cannot be conducted owing to the limited number of cases. For example, in the study of DM combined with different DR stages, there were only two cases of PDR, and neither was combined with dry nor wet AMD; therefore, the study of PDR combined with dry and wet AMD cannot be counted. The stages of AMD include early, middle, and late stages, such as GA and wet AMD. The stages of DR included NPDR and PDR, and NPDR included mild, moderate, and severe NPDR. It is very difficult to correlate each stage of DR to each stage and type of AMD individually; therefore, epidemiological research or meta-analysis with a larger sample size is needed.

In conclusion, to our knowledge, this is the first epidemiological population-based study to discuss the relationship between DR and AMD in the northern mainland China. This study confirmed that the prevalence of dry AMD in DM complicated with DR was increased, and DR was a risk factor for dry AMD.

For patients with DM, how to screen for DR early and how to reduce or delay the occurrence of DR to effectively reduce the prevalence of AMD is an important topic. Future research aims to use new screening techniques combined with the in-depth learning artificial intelligence technique, to screen for early DR (62–65). The precise changes in choroidal thickness and structure of DR and AMD may provide evidence for the common pathogenesis of DR and AMD, although it is complex and unclear and needs to be discovered. An animal model of diabetic choroidopathy and the construction of animal models for DR combined with CNV are needed in the future (66). Whether PRP and anti-VEGF therapy can delay AMD occurrence in patients with DR should be investigated. Larger and longitudinal population-based epidemiological studies are needed to determine the exact relationship between the different stages of DR and AMD.

## Data Availability Statement

The datasets presented in this study can be found in online repositories. The names of the repository/repositories and accession number(s) can be found in the article/supplementary material.

## Ethics Statement

The studies involving human participants were reviewed and approved by Beijing Tongren Hospital. The patients/participants provided their written informed consent to participate in this study.

## Author Contributions

ZY, ZJinq, and WW contributed to the interpretation of data and drafting of the report. WW contributed to the study design and review. All authors contributed to the article and approved the submitted version.

## Conflict of Interest

CS was employed by Kailuan Group. The remaining authors declare that the research was conducted in the absence of any commercial or financial relationships that could be construed as a potential conflict of interest.

## Publisher's Note

All claims expressed in this article are solely those of the authors and do not necessarily represent those of their affiliated organizations, or those of the publisher, the editors and the reviewers. Any product that may be evaluated in this article, or claim that may be made by its manufacturer, is not guaranteed or endorsed by the publisher.
